# Antigenic analysis of the HIV-1 envelope trimer implies small differences between structural states 1 and 2

**DOI:** 10.1016/j.jbc.2022.101819

**Published:** 2022-03-10

**Authors:** Evan M. Cale, Jefferson I. Driscoll, Myungjin Lee, Jason Gorman, Tongqing Zhou, Maolin Lu, Hui Geng, Yen-Ting Lai, Gwo-Yu Chuang, Nicole A. Doria-Rose, Walther Mothes, Peter D. Kwong, John R. Mascola

**Affiliations:** 1 Vaccine Research Center, National Institute of Allergy and Infectious Diseases, National Institutes of Health, Bethesda, Maryland, USA; 2 Department of Microbial Pathogenesis, Boyer Center for Molecular Medicine, Yale University School of Medicine, New Haven, Connecticut, USA; 3 Department of Cellular and Molecular Biology, University of Texas Health Science Center, Tyler, Texas, USA

**Keywords:** broadly neutralizing antibody, conformational change, flow cytometry, glycoprotein structure, HIV-1, intermediate conformations, smFRET, SOSIP, ABR, antibody binding ratio, bnAbs, broadly neutralizing antibodies, DMSO, dimethyl sulfoxide, EM, electron microscopy, Env, envelope glycoprotein, Fab, fragment antigen binding, MFIs, median fluorescence intensities, RMSD, root-mean-square deviation, sCD4, soluble CD4, smFRET, single-molecule Förster resonance energy transfer, V1, variable loop 1, V2, variable loop 2

## Abstract

The conformationally dynamic HIV-1 envelope trimer (Env) is the target of broadly neutralizing antibodies (bnAbs) that block viral entry. Single-molecule Förster resonance energy transfer (smFRET) has revealed that HIV-1 Env exists in at least three conformational states on the virion. Prior to complete host–receptor engagement (State 3), Env resides most prevalently in the smFRET-defined State 1, which is preferentially recognized by most bnAbs that are elicited by natural infection. smFRET has also revealed that soluble trimers containing prefusion-stabilizing disulfide and isoleucine-to-proline substitutions reside primarily in State 2, which is a required intermediate between States 1 and 3. While high-resolution Env structures have been determined for States 2 and 3, the structure of these trimers in State 1 is unknown. To provide insight into the State 1 structure, here we characterized antigenic differences between smFRET-defined states and then correlated these differences with known structural differences between States 2 and 3. We found that cell surface–expressed Env was enriched in each state using state-enriching antibody fragments or small-molecule virus entry inhibitors and then assessed binding to HIV-1 bnAbs preferentially binding different states. We observed small but consistent differences in binding between Env enriched in States 1 and 2, and a more than 10-fold difference in binding to Env enriched in these states *versus* Env enriched in State 3. We conclude that structural differences between HIV-1 Env States 1 and 3 are likely more than 10-fold greater than those between States 1 and 2, providing important insight into State 1.

The HIV-1 envelope glycoprotein (Env), or spike, is critical for virus entry into host CD4+ T cells and is also the target of HIV-1–specific broadly neutralizing antibodies (bnAbs), a number of which have been identified with exceptional neutralization breadth and potency against diverse virus isolates (1, 2, 3, 4, 5, 6). As such, Env remains a focus in the HIV-1 vaccine field as a potential immunogen (1, 7, 8). HIV-1 Env exists on the virion surface as a prefusion trimer of three protomers consisting of gp120 and gp41 heterodimers (9, 10, 11). CD4 binding of Env induces conformational changes that cause the variable loops 1 and 2 (V1V2) of Env to disassemble from the apex of the trimer and reposition to the trimer periphery, a structural transition which exposes the binding site for the coreceptor CCR5 or CXCR4 (4, 9, 12, 13, 14, 15). Coreceptor binding induces additional conformational changes, particularly in the gp41 subunits anchoring the trimer in the membrane, that enable subsequent fusion of the virus and host cell membranes (16, 17, 18).

To study the conformational dynamics of Env on membrane-bound and soluble trimers, single-molecule Förster resonance energy transfer (smFRET) has been used to monitor the relative movement of fluorescently labeled V1 and either V4 or V5 loops on native trimers over time, with or without engagement of trimer by various Env-binding ligands (15). These studies have revealed that unliganded trimers exist predominantly in a low-FRET state (State 1) but can sample two additional conformational states, a high-FRET state (State 2) where the V1 and V4/V5 loops are in closer proximity to one another, and a third state of intermediate-FRET (State 3) (15, 19, 20). CD4 binding triggers the transformation to the conformational intermediate State 2, and saturation of all three protomers with CD4 induces the adoption of a predominantly State 3 conformation (15, 19, 20). In addition to V1 and V4 or V5 labels, labels in V4 and α6-helix of gp41 confirmed these features from a second perspective (6).

smFRET studies have shown that most naturally elicited HIV-1 bnAbs exhibit a preference for binding to Env in State 1, with the notable exception of the gp120-gp41 interface-targeting bnAb PGT151 that prefers binding to, and enriches Env in, State 2 (6, 15). Moreover, the Bristol Myers-Squibb series of small molecule HIV-1 entry inhibitors such as BMS-378806 and BMS-626529/temsavir, whose prodrug form BMS-663068/fostemsavir was approved in 2020 for clinical use by the U.S. Food and Drug Administration, also stabilize and prefer binding to the HIV-1 Env State 1 conformation (6, 15, 21, 22, 23, 24). Recent studies have shown that the soluble cleaved SOSIP trimers used in many structural studies to date, which have engineered disulfide bonds to stabilize the interaction of gp120 and gp41, adopt a State 2 conformation that cannot be altered by coincubation of State 1–enriching ligands; the disulfide “SOS” (A501C/T605C) knock-in mutations were found to be responsible for stabilizing the trimer in State 2 (6). Attempts to visualize State 1 structures of native Env by cryo-electron microscopy (EM) have been hampered by inactivation of these particles, for safety reasons, with aldrithiol-2; this treatment also induced trimers to adopt the State 2 conformation (25). Therefore, the structure of HIV-1 Env in State 1 remains elusive.

Studies of SOSIP-immunized cows have shown that elicited neutralizing antibodies prefer and enrich Env trimers in State 2, consistent with the SOS mutation-mediated enrichment of soluble SOSIPs in this conformational state (26). Because the vast majority of naturally elicited HIV-1 bnAbs prefer to bind to and enrich trimers in State 1, the determination of a State 1 structure continues to be a priority in the field (27). The hypothesis of an additional State 1 for HIV-1 Env as suggested by smFRET has been questioned by several other studies (28, 29, 30). As such, studies on Env conformations independent of smFRET are of great importance to resolve this controversy. Here, we measure antigenic differences of Env enriched in the three smFRET-determined conformational states by antibody binding to estimate the relative structural differences between State 1 and States 2 and 3, which have already determined structures. Our data indicate reproducible differences between these states, although they are smaller than suggested by smFRET.

## Results

### Study design

**Figure 1:**
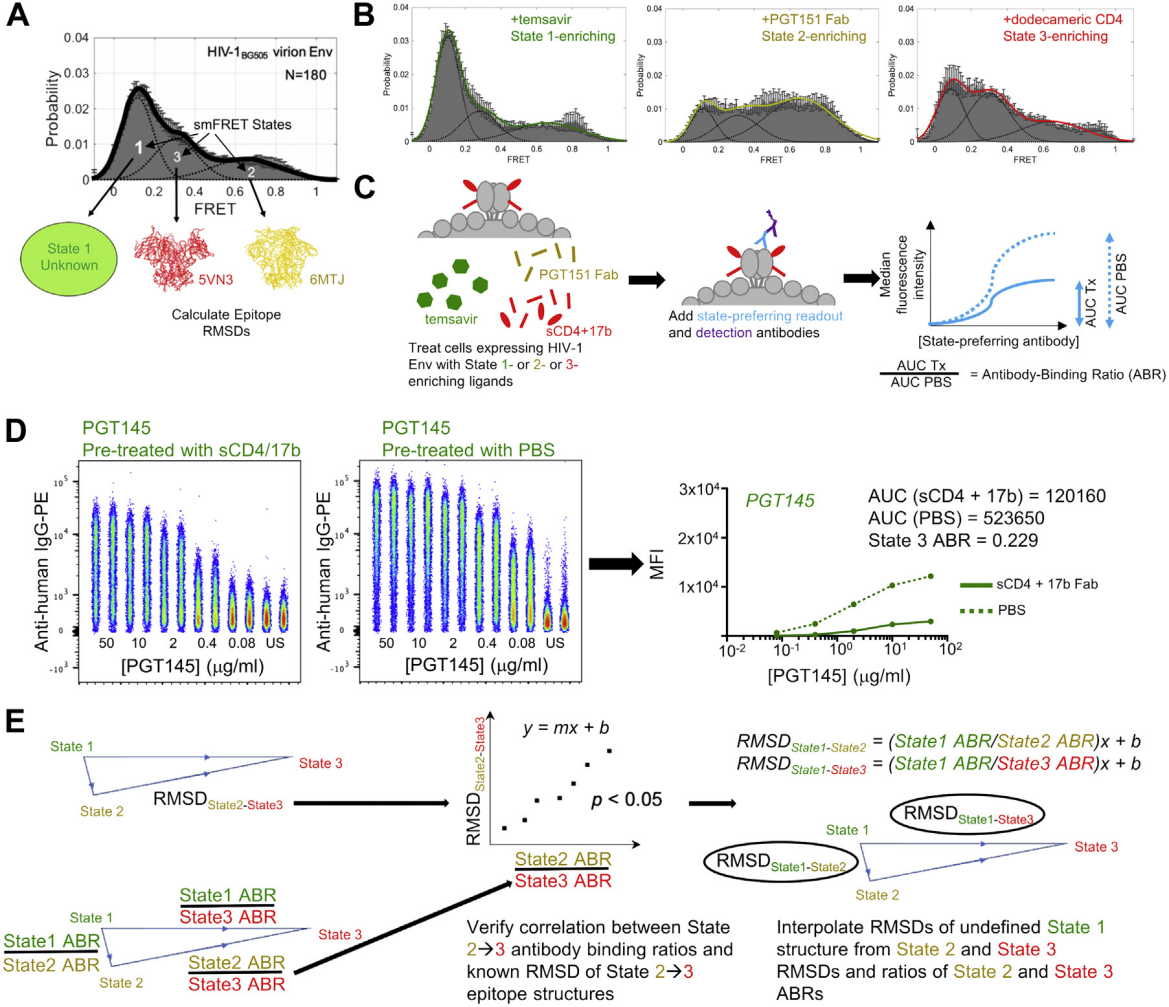
**Interpolating RMSDs between smFRET States 1 and 2 and between States 1 and 3 based on State-preferring antibody binding to HIV-1 Env enriched in different states.***A*, smFRET indicates that the native HIV-1 Env trimer adopts 3 conformational States 1, 2, and 3. Structures of the HIV-1 Env trimer have been determined for the State 2 (PDB 6MTJ, *yellow*) and the State 3 (PDB 5VN3, *red*) conformational states, enabling the calculation of the RMSD of HIV-1 nAb-bound epitope structures in States 2 and 3. The structure of the State 1 conformational state (*green*) remains unsolved. *Green*, *yellow*, and *red* color-coding for States 1, 2, and 3 conditions are consistent throughout the data. *B*, shown are representative smFRET histograms when JR-FL Env trimer is pretreated with State 1-, State 2-, and State 3-enriching ligands BMS-626529/temsavir, PGT151 Fab, and dodecameric CD4, respectively. *C*, binding differences of antibody epitopes in the different smFRET states are determined using a 293T cell line expressing JR-FL native Env trimers that is pretreated with different state-enriching ligands. Serially diluted primary HIV-1 IgGs of known state preference (*blue*) are then incubated with pretreated cells/trimers, and IgG binding is detected with a fluorescently labeled anti-IgG antibody (*purple*). The ratio of the areas under the curve of ligand-treated trimer binding (AUC Tx) to mock-treated trimer binding (AUC PBS) are calculated as the antibody binding ratio (ABR) of the antibody to that particular enriched state. sCD4: soluble CD4. *D*, example raw flow cytometry data showing binding of PE-conjugated anti-human IgG to JR-FL-expressing 293T cells pretreated with soluble CD4 and 17b Fab (*left plot*) or with PBS (*right plot*). Staining was performed in duplicate for each PGT145 antibody concentration shown. Average median fluorescence intensities (MFIs) were plotted against antibody concentrations to obtain area under the curve (AUC), from which ABRs were calculated. These data are also shown in Fig. S2 along with the rest of the entire dataset for pretreatment with soluble CD4/17b Fab. *E*, the RMSDs of State 2- and State 3-enriched bnAb epitopes, as determined from State 2 and 3 structures 6MTJ and 5VN3, respectively, are plotted against the ratios of the ABRs of State 2 and State 3 for the respective bnAbs, and a Pearson correlation is determined. The Pearson equation is used to interpolate the RMSDs of State 1- and State 2-enriched bnAb epitopes and the RMSDs of State 1- and State 3-enriched bnAb epitopes as shown. bnAb, broadly neutralizing antibody; Fab, fragment antigen binding ; RMSD, root-mean-square deviation; smFRET, single-molecule Förster resonance energy transfer.

We used two previously published HIV-1 Env structures in known smFRET-defined States as templates for smFRET State 2 and State 3 structures. For smFRET State 2, we selected a structure of HIV-1 BG505 SOSIP.664 prefusion Env trimer in complex with the small-molecule HIV entry inhibitor BMS-378806 and human antibodies 3H109L, containing heavy and light chain intermediates of the glycan-V3-directed PGT121 bnAb lineage, and the gp120-gp41 interface-directed bnAb 35O22 (PDB 6MTJ) (Fig. 1*A*) (31). For smFRET State 3, we chose a cryo-EM model of HIV-1 B41 SOSIP.664 in complex with a dodecameric form of soluble CD4 (sCD4) and the fragment antigen binding (Fab) of the CD4-inducible antibody 17b (PDB 5VN3) that are known to enrich State 3 (Fig. 1*A*) (14). From these two structures, we were able to determine the root-mean-square deviation (RMSD; defined as the average distance between atoms of two superimposed proteins) of various known HIV-1 bnAb epitopes in State 2 and State 3 (Fig. 1*A*).

To determine antigenic differences between smFRET-defined States 1, 2, and 3, we made use of three ligands that preferentially enrich HIV-1 trimers in distinct States: the small-molecule HIV-1 entry inhibitor BMS-626529/temsavir that enriches trimers in State 1, PGT151 Fab that enriches State 2, and a cocktail of sCD4 and 17b Fab that enriches State 3 in the same manner as dodecameric CD4 (Fig. 1*B*) (6, 15). 293T cells were transfected with plasmids encoding JR-FL.E168K.dCT (herein referred to as “JR-FL”) Env glycoprotein and furin such that the cells expressed fully cleaved JR-FL trimers on the cell surface (Fig. 1*C*). The presence of properly folded trimers was confirmed by assessing cell surface Env binding to the quaternary-dependent HIV-1 bnAb PGT145 (Fig. S1, *A* and *B*) (32). Protomer cleavage was confirmed by detecting binding to the cleavage-specific bnAb PGT151 (Fig. S1, *A* and *B*) (33). Failure to bind to the respiratory syncytial virus–specific antibody palivizumab indicated little nonspecific binding in the assay (Fig. S1, *A* and *B*) (34, 35). Trimer-expressing cells were then treated with BMS-626529/temsavir, PGT151 Fab, or sCD4+17b Fab to enrich the trimers in States 1, 2, or 3, respectively. Serially diluted antibodies of known smFRET-defined State preference and epitope specificity were then added, and antibody binding was detected with an anti-human-IgG detection antibody conjugated with the fluorophore phycoerythrin and measured by flow cytometry. The antibody binding ratio (ABR) was then calculated by taking the ratio of the area under the curve (AUC) of antibody binding under conditions of the State-enriching ligand treatment (AUC Tx) and in the absence of the ligand (PBS treatment, AUC PBS) (Fig. 1, *C* and *D*).

While the structure of smFRET-defined State 1 remains unknown, we hypothesized that we could infer how much State 1 structurally differs from States 2 and 3 from the differences in HIV-1 bnAb binding to trimers enriched in the three different States. The relative antibody binding differences between two States were determined by calculating the ratios of ABRs to the two given States (Fig. 1*E*). The RMSDs of epitopes for different HIV-1 bnAbs between States 2 and 3 were determined from the State 2 and 3 structures and plotted against the ratio of ABRs of State 2 and State 3 binding for each antibody, and a linear relationship between the data was determined (Fig. 1*E*). The RMSDs of bnAb epitopes between States 1 and 3 and between States 1 and 2 were then interpolated from the Pearson regression equation (Fig. 1*E*).

### Enrichment of JR-FL trimers in State 3 dramatically reduces binding of State 1- and State 2-preferring antibodies and improves binding of a State 3-preferring antibody

**Figure 2:**
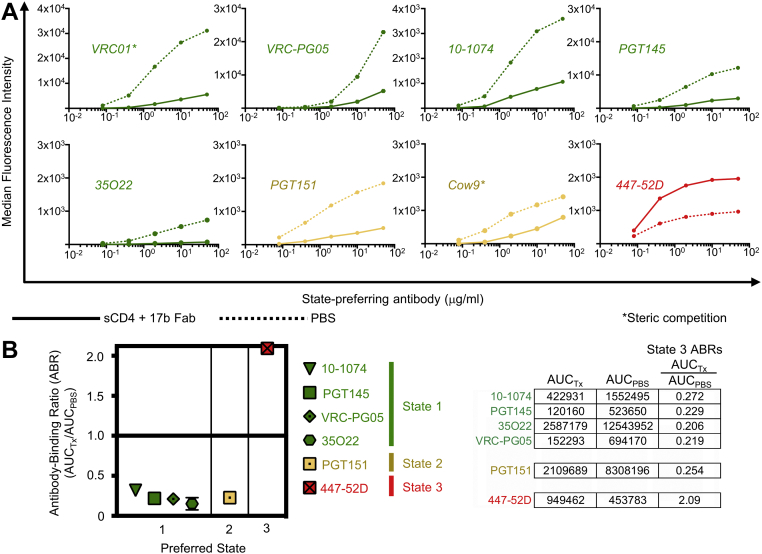
**Enrichment of HIV-1 Env trimers in State 3 using sCD4 and 17b Fab reduced binding of State 1- and State 2-preferring antibodies**. *A*, binding of serially diluted HIV-1–specific bnAb IgGs (various colors) to 293T cell surface–expressed, fully cleaved JR-FL Env trimers pretreated with soluble CD4 and 17b Fab (*solid lines*) or with PBS (*dotted lines*) are shown. The *asterisk* indicates that IgG binding was competed by presence of the indicated Fab used for trimer pretreatment. *B*, ratios of area under the curve of sCD4/17b-treated trimers (AUC_Tx_) to area under the curve of mock-treated trimers (AUC_PBS_) are plotted for each bnAb. Shown are mean ± SD of at least two experiments. At *right* are shown example ABR calculations from a representative experiment. ABR, antibody binding ratio; bnAb, broadly neutralizing antibody; Fab, fragment antigen binding; sCD4, soluble CD4.

To assess the binding of different HIV-1–specific antibodies to trimers enriched in State 3, we used a combination of sCD4 and 17b Fab that induces conformational changes in the trimer structure to adopt a State 3 conformation (Fig. 1*B*). Five HIV-1 antibodies with known State 1 preference were then assessed for binding to trimers in the presence or absence of sCD4+17b: the CD4 binding site–specific bnAb VRC01, the silent face center-targeting bnAb VRC-PG05, glycan-V3–specific 10-1074, glycan-V1V2–specific PGT145, and the gp120:gp41 subunit interface-targeting bnAb 35O22 (Table S1). Enrichment of trimers in State 3 resulted in dramatically reduced binding by all five antibodies (Figs. 2*A* and S2), although reduced binding of VRC01 was attributed to steric clashing between these antibodies and sCD4 (Figs. 2*A* and S3*A*) (36, 37). For the remaining four antibodies that are not sterically impacted by the presence of sCD4 and 17b Fab, we observed a greater than 65 percent decrease in trimer binding to State 3-enriched trimers compared to unenriched trimers (Figs. 2*A* and S2). We observed a similar decrease in binding to two State 2-preferring antibodies, CD4 binding site–specific Cow9, and gp120:gp41 subunit interface-specific PGT151, when trimers were enriched in State 3 (Figs. 2*A* and S2), although reduced Cow9 binding was attributed to steric competition with sCD4, as determined from the structural overlap of sCD4 and a clonal relative of Cow9, Cow1 when bound to gp140 (Figs. 2*A* and S3*A*) (36, 38). In contrast, the State 3-preferring antibody 447-52D, which targets the V3 loop of gp120, exhibited a greater than 100 percent increase in binding to State 3-enriched trimers compared to PBS-treated trimers (Fig. 2*A*).

We next calculated the ABRs for each antibody that was not sterically blocked by the State 3-enriching ligands sCD4 and 17b Fab. We found that State 1-preferring Abs 10-1074, PGT145, VRC-PG05, and 35O22 and State 2-preferring antibody PGT151 had similar ABRs ranging from 0.21 to 0.27, indicating reduced binding due to State 3 enrichment, while 447-52D had an ABR above 2.0, indicating greatly increased binding due to State 3 enrichment (Fig. 2*B*).

### Enrichment of HIV-1 trimers in State 2 causes a minor but consistent reduction of State 1- and State 3-preferring antibodies

**Figure 3:**
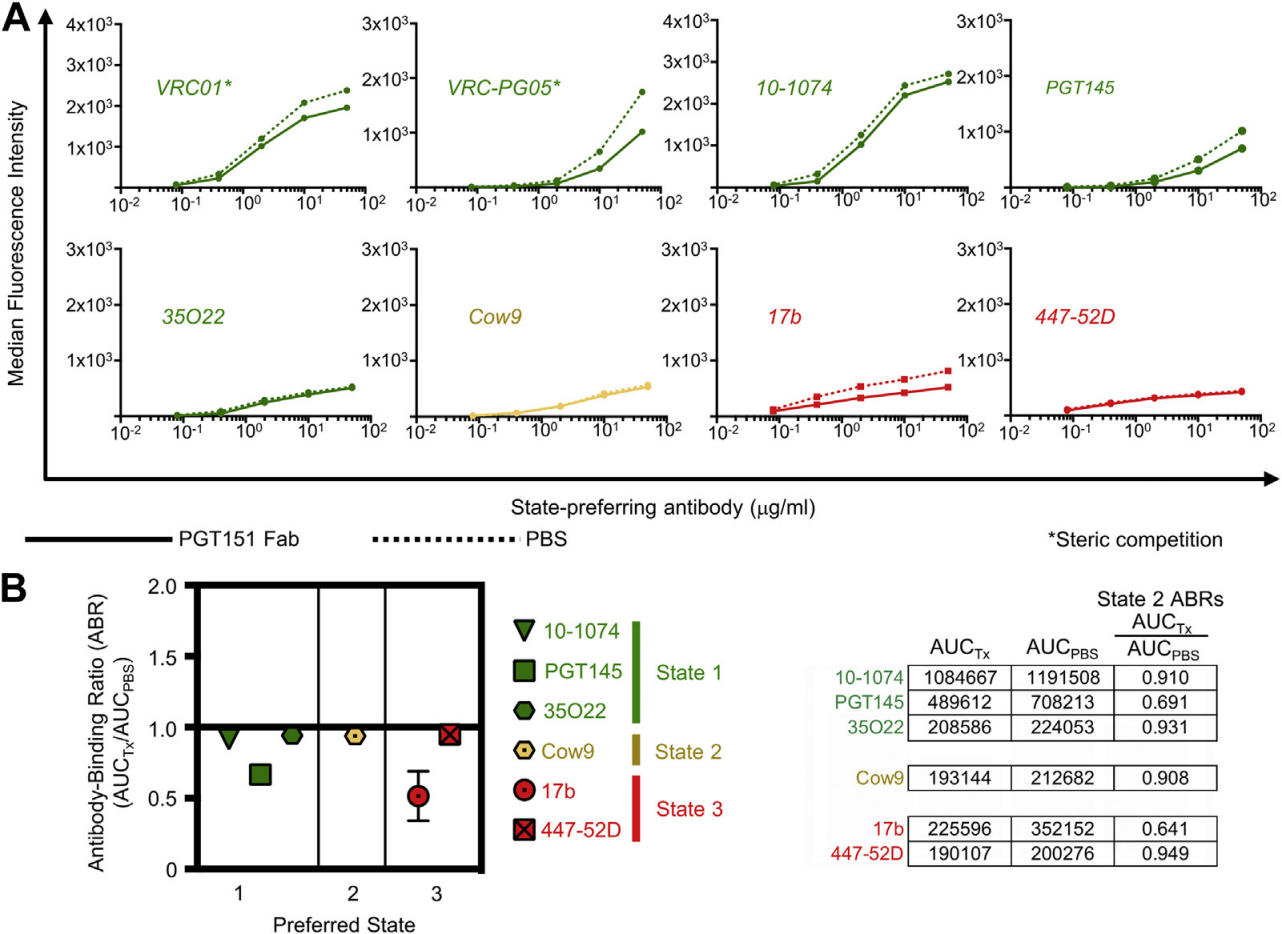
**Enrichment of State 2 using PGT151 Fab causes a minor to moderate reduction in binding of State 1- and State 3-preferring antibodies**. *A*, binding of serially diluted HIV-1–specific bnAb IgGs (various colors) to 293T cell surface–expressed, fully cleaved JR-FL Env trimers pretreated with PGT151 Fab (*solid lines*) or with PBS (*dotted lines*) are shown. The *asterisk* indicates that IgG binding was competed by presence of the indicated Fab used for trimer pretreatment. *B*, ratios of area under the curve of PGT151-treated trimers (AUC_Tx_) to area under the curve of mock-treated trimers (AUC_PBS_) are plotted for each bnAb. Shown are mean ± SD of at least two experiments. At *right* are shown example ABR calculations from a representative experiment. ABR, antibody binding ratio; bnAb, broadly neutralizing antibody; Fab, fragment antigen binding.

To assess HIV-1 antibody binding to trimers enriched in smFRET-defined State 2, we used PGT151 Fab for State 2 enrichment (Fig. 1*B*). Abs VRC01 and VRC-PG05 exhibited slightly reduced binding that can be attributed to some steric competition with PGT151 (Figs. 3*A*, S3*B*, and S4) (37, 39, 40); however, two other State 1-preferring Abs, 10-1074 and 35O22, with no known steric clashing with PGT151 both exhibited a slight but negligible reduction (0.06–0.08 percent) in binding to State 2-enriched JR-FL Env compared to unenriched Env, while PGT145 exhibited a greater loss of binding by 33 percent (Figs. 3*A* and S4). The same slightly reduced binding observed for 10-1074 and 35O22 was observed for the State 2-preferring antibody Cow9 and the State 3-preferring antibody 447-52D when JR-FL was enriched in State 2 (Figs. 3*A* and S4). In contrast, State 3-preferring antibody 17b exhibited a 50 percent decrease in binding to State 2-enriched trimers compared to unenriched trimers (Figs. 3*A* and S4). ABRs were calculated for all Abs not predicted to sterically compete with PGT151 Fab for trimer binding and were found to range from 0.64 to 0.95, indicating that State 2 enrichment did not have as great an impact on antibody binding compared to State 3 trimer enrichment (Fig. 3*B*).

### Enrichment of HIV-1 trimers in State 1 has minimal effect on State 1- and State 2-preferring antibodies but reduces binding of State 3-preferring antibodies

**Figure 4:**
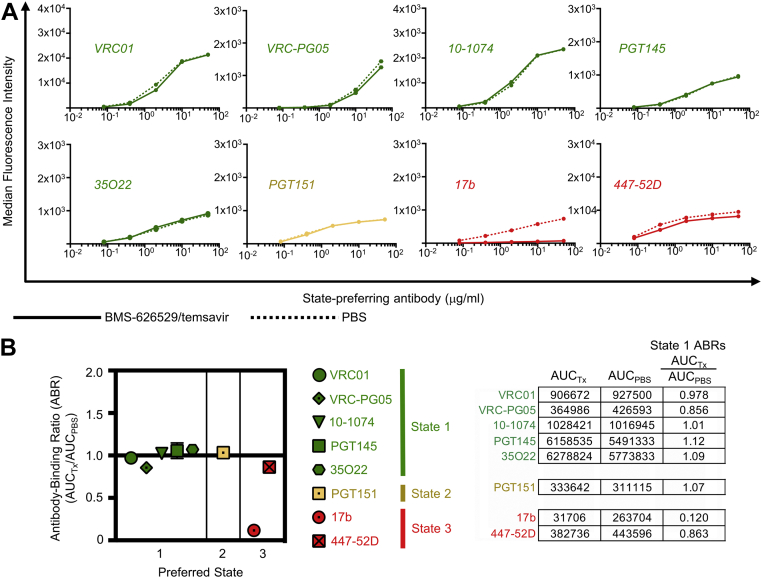
**Enrichment of State 1 using BMS-626529/temsavir has minimal effect on binding of State 2-preferring antibodies but reduces binding of State 3-preferring antibodies**. *A*, binding of serially diluted HIV-1–specific bnAb IgGs (various colors) to 293T cell surface–expressed, fully cleaved JR-FL Env trimers pretreated with BMS-626529/temsavir (*solid lines*) or with PBS (*dotted lines*) are shown. *B*, ratios of area under the curve of BMS-626529/temsavir-treated trimers (AUC_Tx_) to area under the curve of mock-treated trimers (AUC_PBS_) are plotted for each bnAb. Shown are mean ± SD of at least two experiments. At *right* are shown example ABR calculations from a representative experiment. ABR, antibody binding ratio; bnAb, broadly neutralizing antibody; Fab, fragment antigen binding; sCD4, soluble CD4.

Finally, we assessed HIV-1 nAb binding to trimers enriched in smFRET-defined State 1 by pretreatment with BMS-626529/temsavir (Fig. 1*B*). Of all State 1-preferring Abs assessed, binding was minimally affected by State 1 enrichment (Figs. 4*A* and S5). The same was observed for State 2-preferring PGT151 and State 3-preferring 447-52D binding to State 1-enriched HIV-1 Env (Figs. 4*A* and S5). In contrast, binding of 17b was dramatically reduced by almost 90 percent when trimers were enriched in State 1 (Figs. 4*A* and S5). ABRs were calculated for all eight antibodies tested for trimer binding and ranged from 0.86 to 1.12 for State 1- and State 2-preferring Abs and 0.12 for State 3-preferring 17b (Fig. 4*B*). Taken together, our cell surface binding results indicate that States 1 and 2 are antigenically similar to each other and are antigenically distinct from State 3.

### Reduced antibody binding due to State enrichment correlates significantly with greater HIV-1 nAb epitope structural differences between different states

**Figure 5:**
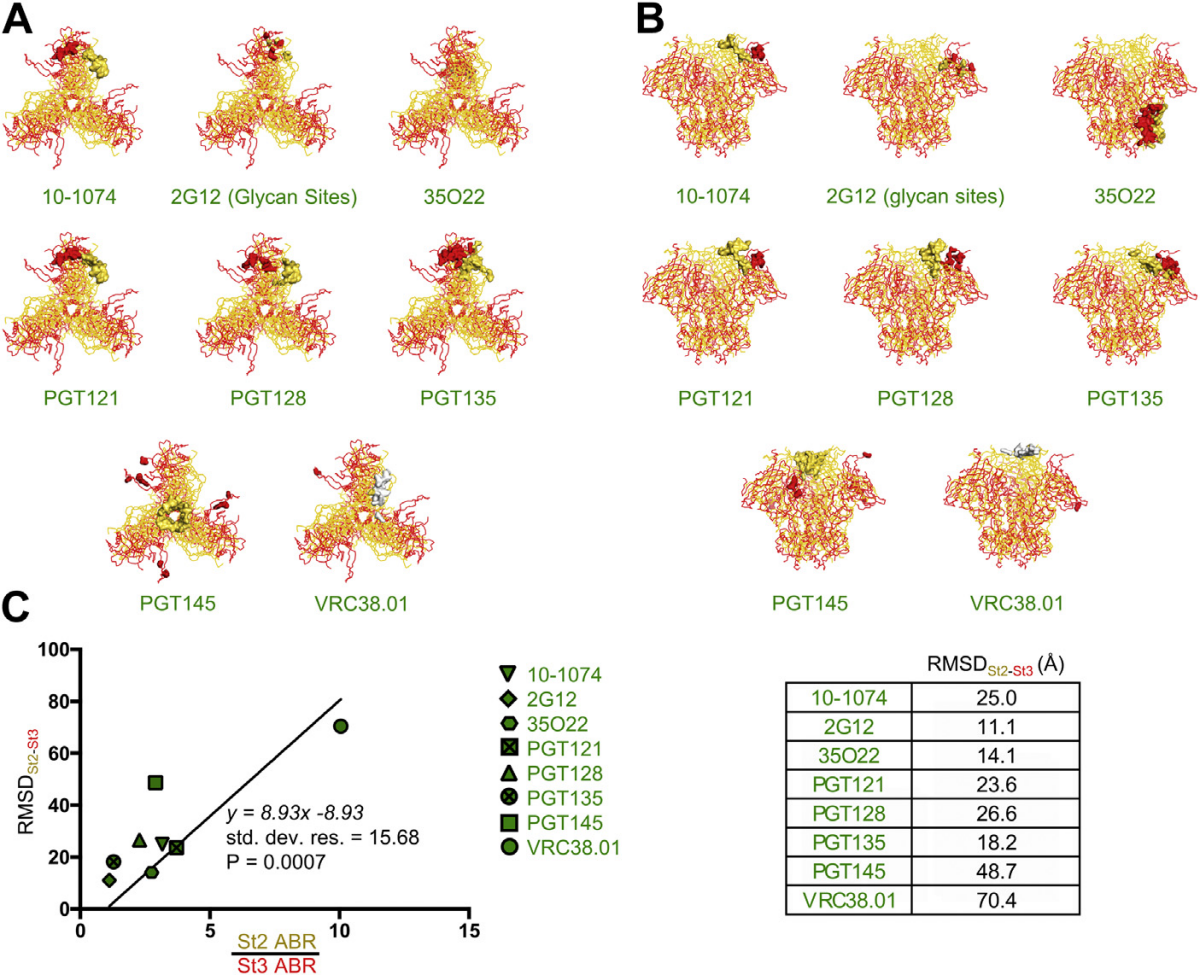
**Reduced antibody binding affinity to HIV-1 Env enriched in different states correlates significantly with higher RMSDs between nAb epitope sites in the different states.***A*, ribbon diagrams of the HIV-1 Env structure enriched in State 2 (*yellow*) and State 3 (*red*) from structures 6MTJ and 5VN3, respectively, are shown as aligned by the entire Env structure as viewed from the top. The epitope sites of different state-preferring antibodies are indicated by the color-coded space-filling models. While only one residue for the VRC38.01 epitope was indicated in the 6MTJ structure, the entire epitope is indicated in the white space-filling model. *B*, ribbon diagrams of the HIV-1 Env structure as viewed from the side. *C*, the RMSD of different nAb-bound epitope sites (indicated in different symbols) in State 2 and State 3, as determined from solved structures 5VN3 and 6MTJ and shown in the table to the *right*, is plotted against the ratio of State 2 ABRs and State 3 ABRs measured for different nAbs in the binding assay, and a Pearson correlation was calculated when forcing the *x* and *y* intercepts through (1,0). ABR, antibody binding ratio; RMSD, root-mean-square deviation.

We examined State 2 structure 6MTJ and State 3 structure 5VN3 to explore conformational differences of different HIV-1 nAb epitopes in these two States. Because 2G12 makes exclusively glycan contacts on the high-mannose patch of gp120, we determined conformational differences between the N-linked glycosylation sites as found in State 2 and 3 structures. For VRC38.01, only residue 130 was resolved in the 5VN3 structure, and the positional difference of this residue was used to calculate the RMSD of the VRC38.01 epitope. When the State 2 and State 3 structures were aligned by Env in its entirety, as opposed to gp41 alone, we found that HIV antibody epitope RMSDs between State 2 and State 3 ranged from as little as 11 Å (2G12) to 70 Å (VRC38.01) (Fig. 5, *A*–*C*). Because we were able to determine antibody binding differences to both State 2- and State 3-enriched trimers for eight HIV-1 antibodies (10–1074, 2G12, 35O22, PGT121, PGT128, PGT135, PGT145, and VRC38.01) (Figs. 2, 3, S2, S4, and S6), we plotted the RMSDs between each of these antibody epitopes in States 2 and 3 against the ratio of ABRs of State 2 and State 3 and forced the *x-* and y-intercepts of the resulting Pearson equation through 1 and 0, respectively. We found that lower RMSDs between epitopes in State 2 and State 3 correlated significantly (*p* = 0.0007) with lower ABR ratios (indicating lower differences in binding to State 2- and State 3-enriched trimers) (Fig. 5*C*). When State 2 and State 3 structures were aligned by gp41 alone, we determined a similar linear relationship (*p* = 0.005) between RMSDs between States 2 and 3 and binding ratios of antibodies between States 2 and 3 (Fig. S7, *A*–*C*). Therefore, we hypothesized that RMSDs between epitopes in State 1 and State 3 and between State 1 and State 2 could be interpolated from the linear relationship between epitope RMSDs between State 2 and State 3 and the ABR ratios between State 2 and State 3 (Fig. 1*E*).

We observed that the linear relationship between the ratios of State 2 and State 3 ABRs to the State 2–State 3 RMSDs appeared to be driven by the outlier VRC38.01, which exhibited a much higher ratio of State 2 and State 3 ABRs as well as higher epitope RMSDs between State 2 and State 3 compared to other antibodies. We re-assessed the data by removing the VRC38.01 outlier and found the correlation between State 2–State 3 epitope RMSDs and ABR ratios remained significant, both when State 2/State 3 structures were aligned by the entire trimer (*p* = 0.0049) and by gp41 alone (*p* = 0.0096) (Fig. S8*A*).

### Based on known structures of State 2 and State 3 HIV-1 Env and antibody binding differences of various HIV-1 Abs to State 1-, 2-, and 3-enriched Env, we predict overall epitope RMSDs between states 1 and 2 to be around 2 Å and between states 1 and 3 to be around 30 Å

**Figure 6:**
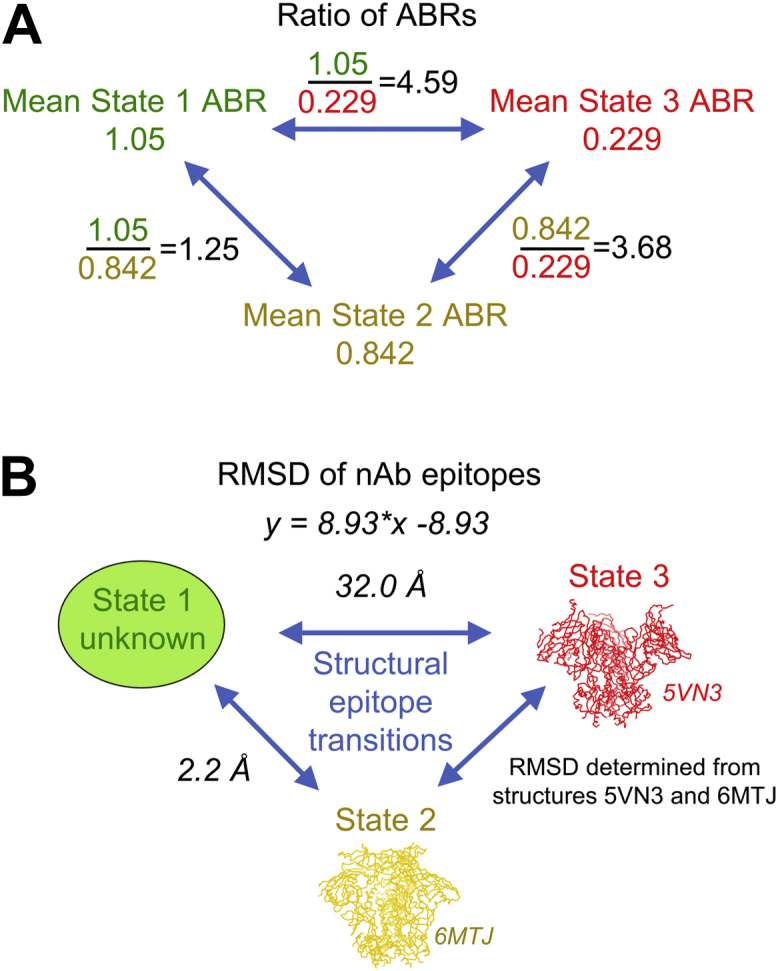
**Based on ratios of ABRs and available structural data, we predict State 1-preferring antibody epitope RMSDs between States 1 and 3 to be about 30 Å and RMSDs between States 1 and 2 to be about 2 Å**. *A*, the ratios of ABRs between State 1 and State 2, between State 1 and State 3, and between State 2 and State 3 were calculated for multiple antibodies using the HIV-1 Env cell surface staining assay. Shown in *black font* are the ratios of the mean of ABRs for 10-1074, 35O22, and PGT145, the three bnAbs from which we could calculate ABRs to trimers stabilized by each of the three states. *B*, the mean RMSDs (*italicized font*) of nAb epitope sites between States 1 and 2 and between States 1 and 3 were calculated from the ratios *x* of the measured ABRs shown in panel A and the Pearson correlation equation (shown) derived in Figure 5*B*, where *y* is the RMSD, and *x* is the ratio of ABRs. ABR, antibody binding ratio; RMSD, root-mean-square deviation.

We were able to determine ABRs for all three smFRET-defined States for three antibodies, 10-1074, PGT145, and 35O22. Therefore, we calculated the ratios of ABRs between State 1 and 3, between State 1 and 2, and between State 2 and 3 for each antibody (Fig. S9*A*). State 1/2 ABR ratios (range: 1.1–1.6) were smaller than State 1/3 ABR ratios (range: 3.2–7.0) and State 2/3 ABR ratios (range: 2.9–6.2) for each antibody, reflecting the relative similarity in antibody binding to States 1 and 2 and the relatively greater difference in binding between each of these States and State 3. We then interpolated epitope RMSDs between States 1 and 3 and between States 1 and 2 from the Pearson regression equation derived in Figure 5*C* (Fig. S9*B*). By this calculation, we determined that the 10-1074 epitope RMSD between States 1 and 3 was approximately 20 Å, and the epitope RMSD between States 1 and 2 was approximately 1 Å (Fig. S9*B*). For PGT145, the RMSD between States 1 and 3 was approximately 34 Å and that between States 1 and 2 was about 5 Å (Fig. S9*B*). Finally, we determined the 35O22 epitope RMSD between States 1 and 3 to be about 54 Å, and the epitope RMSD between States 1 and 2 to be about 1 Å (Fig. S9*B*). When aligned by gp41, the same pattern of higher RMSDs between States 1 and 3 epitopes compared to States 1 and 2 epitopes, and the magnitude of the RMSDs was slightly higher (ranging 23–63 Å between States 1 and 3 and 1–6 Å between States 1 and 2) (Fig. S7*E*). When averaging the RMSDs of 10-1074, 35O22, and PGT145 between States 1 and 3 and between States 1 and 2 when structures were aligned by the entire Env trimer, we predicted an overall epitope RMSD between States 1 and 3 to be approximately 32 Å and RMSD between States 1 and 2 to be around 2 Å (Fig. 6, *A* and *B*). When structures were aligned by gp41, these RMSDs were slightly higher (about 38 Å for States 1 and 3 epitope RMSDs and about 3 Å for States 1 and 2 epitope RMSDs) (Fig. S7, *D* and *E*).

When the outlier VRC38.01 was removed from the data (Fig. S8*A*), we re-calculated the epitope RMSDs between States 1 and 3 and between States 1 and 2 for State 1-preferring Abs. When the structures 5VN3 and 6MTJ were aligned by the entire Env trimer, the overall epitope RMSD between States 1 and 3 were approximately 50 Å, while the RMSD between States 1 and 2 were about 3.4 Å (Fig. S8*B*). When the structures were aligned by gp41, predicted RMSDs between States 1 and 3 and between States 1 and 2 were approximately 70 Å and 5 Å, respectively (Fig. S8*B*).

## Discussion

To date, the structure of HIV-1 Env trimers in smFRET-defined State 1 remains unsolved, yet this remains a priority in the HIV vaccine field for two major reasons (27). First, the vast majority of HIV-1 bnAbs isolated from HIV-1+ donors to date exhibit a binding preference for State 1, with the notable exception of PGT151 (6, 15). Therefore, it is assumed that HIV-1 Env trimers in this State 1 conformation elicit these bnAbs during natural infection. Secondly, in immunization studies using SOSIP trimers in cows, induced antibodies were found to prefer Env in the smFRET-defined State 2 conformation (6, 26). smFRET studies of SOSIP trimers have shown that the SOS-stabilizing mutations stabilize the trimer in State 2, and this conformation is likely eliciting antibodies that prefer this State (6). While it remains to be determined if State 2-preferring bnAbs are consistently inferior to State 1-preferring bnAbs with regard to breadth and potency, the distinct conformational preference of naturally elicited *versus* SOSIP immunogen-elicited bnAbs, as shown by smFRET, suggests real structural differences between Env conformations in the soluble native-like trimer and in Env presented naturally on the surface of HIV-1 virions and infected cells.

High-resolution structures of full-length HIV-1 Env in complex with State 1-preferring and -enriching antibodies have been published, but questions remain as to if these structures truly represent HIV-1 Env in smFRET State 1. In one study, PGT151- and PGT145-bound full-length AMC011 Env trimers were purified from Env-/furin-transfected and solubilized 293F cells, and their structures and antigenic characteristics were compared (28). Single-particle cryo-EM structures of each full-length trimer displayed on the resulting bicelle preparations appeared to be very similar, with Cα RMSDs between gp120 domains and between gp41 domains each being approximately 1 Å. However, such preparations of detergent-solubilized full-length Env have not been studied by smFRET to determine if they do exhibit State 1-predominant profiles. Indeed, the structural stability of HIV-1 Env has been found to be highly sensitive to changes in cholesterol and protein compositions (41). While the authors do include cholesteryl hemisuccinate as a cholesterol substitute in their preparations, it remains to be demonstrated that the full-length Envs in these solubilized cell preparations properly partition into lipid rafts as Env would normally do on the virion surface and whether the cholesterol hemisuccinate adequately mimics natural cholesterol found on virion surfaces. Similar caveats apply to another study in which PG16-bound full-length 92UG037.8 Env was purified from detergent-solubilized 293T cells (29). There, the cryo-EM structure was remarkably similar to that of soluble BG505 SOSIP.664. Again, the highly similar structures may be due to the enrichment of smFRET-defined State 2 as a result of the detergent solubilization of the full-length Env, which has yet to be determined by smFRET in this setting. Current work investigating the effects of cholesterol composition on Env conformations by smFRET is ongoing.

Structural studies of HIV-1 Env in its prefusion conformation prior to CD4 binding (State 1) have been hampered to date for a number of reasons. The SOSIP trimers that have been the tool of choice in the field for crystal structure and EM studies were found to be stabilized in the State 2 conformation, a phenomenon attributed to the disulfide mutations that covalently attach gp120 to gp41 and not the isoleucine-to-proline substitutions in gp41 that prevent trimers from adopting their terminally opened conformational state (6). Therefore, all structures published to date using the SOSIP platform are believed to be in smFRET State 2. Recent cryo-EM studies have demonstrated that membrane-embedded trimers with the SOSIP modifications also adopt a State 2 conformation, but unlike soluble SOSIP trimers, these membrane-displayed trimers are capable of transitioning to States 1 and 3 with the addition of State 1- and State 3-enriching ligands (27). While this is a major advance in the pursuit of a State 1 structure, aldrithiol-2 inactivation of virus particles necessary for safety purposes remains a barrier, as this process also induces trimers to adopt a State 2 conformation (25).

Here, we estimated the structural differences between smFRET-defined State 1 and the known structures of States 2 and 3 by characterizing the relative binding affinities of various HIV-1 bnAbs for Env enriched in the three different states by state-preferring ligands. Because SOSIPs have been shown to be stabilized in a State 2 conformation, we evaluated binding of bnAbs to JR-FL Env that was engineered to be expressed on the surface of 293T cells, which are believed to display Env on a lipid bilayer much as it would be expressed in its native conformation on virions. Additionally, we co-transfected cells with a plasmid encoding furin to ensure maximal cleavage of gp160 into its gp120 and gp41 components, again mimicking the natural processing of Env by host furin during protein folding in the Golgi. We confirmed that our cell surface–expressed JR-FL Env was capable of binding to the trimer-specific bnAb PGT145 and the cleavage-dependent bnAb PGT151. For these reasons, we believe that cell-surface display of Env is an appropriate tool for evaluating the antigenicity of the different conformational states of Env as would be sampled on native virions.

A major premise in this analysis is that antigenic differences between smFRET-defined conformational states would be proportional to overall structural differences, with regards to RMSD, between these States. While RMSD, in the structural sense, and antibody binding affinity are two very different things that we did not expect to have a relationship *a priori*, we examined if there was any relationship between the two based on our measurements. Therefore, we correlated the RMSD of different bnAb epitopes in States 2 and 3, as determined by known State 2 and State 3 structures, with measured antigenic differences of trimers enriched in these two States. These correlations proved to be statistically significant when the State 2 and 3 structures were aligned by the entire Env trimer as a whole, as well as when the trimers were aligned by gp41 alone. The statistical significance of these correlations held when we removed an outlier, VRC38.01, that appeared to skew the linear relationship between the two variables. We believe that the significant correlations determined by both of these two alignment methods attests to the robustness of our method to estimate structural differences of State 1 from States 2 and 3 by measuring relative binding affinities of bnAbs for the three different states.

We have found that enriching trimers for State 1 with BMS-626529/temsavir did not result in enhanced binding to JR-FL by State 1-preferring bnAbs. This is likely because the Tier 2 strain JR-FL already exhibits a strong State 1 conformation, more than the lab-adapted strain NL4-3 that shows more conformational plasticity in response to co-incubation with sCD4 (enrichment in State 2) or sCD4 in combination with 17b (enrichment in State 3) (15). Therefore, it is probable that any further enrichment of a trimer already predominantly in State 1 would make little difference with regard to trimer binding by bnAbs that prefer this conformational state.

The observed binding of PGT145 to Env enriched in State 3 by sCD4 and 17b was unexpected. PGT145 is known to exhibit a dependence on a quaternary epitope at the apex of the trimer by contacting all three protomers (32, 42). When the trimer is enriched in State 3, the three protomers come apart and move to the periphery of the trimer, effectively removing the epitope for PGT145. The low-level binding of PGT145 to State 3-enriched trimer may be attributable to background, nonspecific binding of the antibody to JR-FL-expressing cells, although this is minimized by thorough washing of the cells between each step in the cell surface binding assay. Some binding may also be attributable to some JR-FL trimers still being able to sample State 1 in the presence of State 3-enriching ligands (Fig. 1*B*) (15). Nevertheless, the binding activity observed is minimal and the lowest among the State 1-preferring bnAbs tested, with the exception of 35O22, which exhibits nearly complete knock-down in binding to State 3-enriched trimers.

The only antibody that exhibited notably increased binding to JR-FL trimers after enrichment with any of the State-preferring ligands was 447-52D, which bound to trimers twice as strongly when enriched in State 3 by preincubation of sCD4 and 17b Fab. 447-52D is a neutralizing antibody of narrow neutralization breadth against neutralization-resistant, tier 2 isolates that targets the V3 loop that is sequestered when the trimer is in the prefusion state (40, 43, 44, 45). This epitope is known to be fully exposed on the trimer surface when Env is triggered for fusion by CD4 and CCR5/CXCR4 coreceptor binding. Therefore, our data suggest that only upon full CD4 saturation and “opening” of the trimer with the CCR5/CXCR4 coreceptor binding site–targeting antibody 17b would dramatically enhance the accessibility of this epitope.

From our analysis, we predict that structural differences between bnAb epitopes on the Env trimer in States 1 and 2 are relatively small, on the order of 1 to 5 Å, when compared to Env in States 1 and 3, where we predict structural RMSDs to be greater than 20 Å. It remains unknown how a structural deviation of 1 to 5 Å translates to differences in bnAb elicitation potential, and indeed, these differences may be so small as to not have any bearing on the relative potentials of State 1 *versus* State 2 immunogens in inducing bnAbs. First, the preference for State 2 conformation over State 1 does not automatically predispose an antibody to inferior breadth or potency, as evidenced by the isolation of the strong State 2-enriching antibody PGT151 (6, 15), whose coverage of 66 percent of a 117-virus panel at a median IC50 of 0.008 qualify it as a truly broad and potent neutralizing antibody (33). Moreover, immunization of cows with SOSIP trimers has elicited bnAbs with greater than 70 percent neutralization breadth against 117 viruses representing globally distributed clades, indicating that State 2-enriched SOSIP trimers are indeed capable of eliciting bnAbs (6, 26). It remains to be determined if these results will translate to human bnAb elicitation by SOSIPs, as cow antibodies have immunogenetic profiles, including CDRH3s approaching 70 amino acids in length, that are quite different from those of human and nonhuman primates, where such loops are typically 10 to 15 amino acids in length and 35 to 37 amino acids in length at their longest (the V2 apex-directed CAP256-VRC26 lineage) for bnAbs identified to date (46, 47, 48, 49, 50, 51). Current ongoing human clinical trials using SOSIP immunogens will shed light on the potential for State 2 Env to elicit bnAbs in humans.

The results reported here are focused on the Tier 2-neutralization resistant and clade B isolate, JR-FL, a strain we selected due to its extensive use in prior smFRET studies and its favorable expression on the cell surface (6, 15, 20, 25). Different isolates from other clades may behave differently with regard to their conformational plasticity as measured by smFRET and antibody binding. This is particularly true for Tier 1 isolates, as mentioned in the case of NL4-3 above, which is conformationally more open and more accessible to CD4 activation in comparison with Tier 2 JR-FL (15). However, another Tier 2 strain from clade A, BG505, has been extensively evaluated by smFRET in response to preincubation with different State-preferring ligands, and the resultant smFRET histograms are very similar to those observed for JR-FL (20, 25). Additionally, a kinetic and thermodynamic comparison between strains NL4-3, BG505, and JR-FL indicated that the differences in the free energies between the different States were similar for the two Tier 2 strains, which both differed substantially from the Tier 1 isolate NL4-3 (20). For these reasons, we believe the results presented in this work are generalizable with respect to other Tier 2 isolates.

Elucidation of the structure of HIV-1 Env in smFRET-defined State 1 remains an ongoing effort. Meanwhile, we took advantage of a cell surface-Env expression system to measure the relative binding affinities of selected bnAbs to Env enriched in each of the three states with known state-preferring/enriching ligands. We demonstrated that the relative binding affinities of bnAbs to State 2- and State 3-enriched Env correlated significantly with RMSDs of the bnAb epitopes from published State 2 and 3 structures, which supports our hypothesis that relative binding affinities to Env enriched in State 1 compared to other States can be used to approximate structural differences between State 1 and the other two States. While the absolute accuracy of our approximation of State 1 and State 2 epitope RMSDs remains to be proven, we predict that State 1-State 3 epitope RMSDs are more than 10-fold higher than State 1-State 2 epitope RMSDs.

## Experimental procedures

### Antibodies

Antibodies used in this study are available from the NIH HIV Reagent Program (HRP, hivreagentprogram.org) or directly from John R. Mascola (JRM, Vaccine Research Center, NIAID, NIH) or Dennis R. Burton (DRB, Scripps Research Institute). CD4 binding site–specific antibodies included VRC01 (HRP Cat#12033) (52) and Cow9 (DRB) (26). The silent face center–specific mAb VRC-PG05 is available from JRM (39). Glycan-V3-/high mannose patch–specific antibodies used were 10-1074 (HRP Cat#12477) (53), 2G12 (HRP Cat#1476) (54), PGT121 (HRP Cat#12343) (32), PGT128 (HRP Cat#13352) (32), and PGT135 (DRB) (32). V2 apex-specific antibodies used were PGT145 (HRP Cat#12703) (32) and VRC38.01 (JRM) (55). gp120-gp41 interface–specific antibodies used were 35O22 (HRP Cat#12586) (56) and PGT151 (DRB) (33). The linear V3 loop–specific antibody 447-52D (57) and the CD4-induced epitope–specific antibody 17b (58) are both available from the HRP (Cat#4030 and Cat#4091, respectively).

### Cell-surface expression of JR-FL Env

8 × 10^6^ HEK 293T cells were seeded in 150 mm^2^ round tissue culture dishes in 20 ml Dulbecco’s Modified Eagle Medium supplemented with 50% fetal bovine serum and 1X penicillin/streptomycin solution. Twenty-four hours later, cells were transfected with plasmids encoding human furin (huFurin) and JR-FL Env. Briefly, for each dish to be transfected, 50 μg of each plasmid was added to 1 ml Opti-MEM. In a separate tube, for each dish to be transfected, 120 μl TruFect-MAX transfection reagent was added to 1 ml Opti-Mem. TruFect-MAX and plasmid mixtures were combined and allowed to sit at room temperature (20–23 ^°^C) for 20 min, and 2 ml transfection mixture was added to each dish of cells dropwise, covering the entire area of the dish. Cells were incubated for 48 h to allow cell surface expression of JR-FL Env.

### Staining JR-FL–expressing cells enriched for smFRET State 1 with BMS-626529/temsavir

JR-FL/huFurin-transfected cells were collected by removing culture supernatant and dislodging cells from dishes with 1X PBS. Cells were centrifuged twice in 1X PBS for 5 min at 210*g*. Cells were resuspended in 1X PBS and split evenly between two tubes. One aliquot of cells was treated with 10 μM BMS-626529/temsavir/2% dimethyl sulfoxide (DMSO)/PBS, and the other with 2% DMSO/PBS, for 30 min at room temperature. LIVE/DEAD Fixable Violet Dead Cell Stain solution (reconstituted in 50 μl of the DMSO provided with kit) was added to cells at a 1:600 dilution and incubated for 30 min at room temperature. Readout antibodies were serially diluted 5-fold and plated in duplicate in 96-well plates, and BMS-/PBS-treated cells were added to antibodies for a final antibody dilution series ranging 50 to 0.08 μg/ml with a pair of wells containing PBS instead of antibody. For wells stained with 17b antibody, 400 nM sCD4 was added to each well. Plates were incubated for 30 min at room temperature, followed by 3 washes in wash buffer (10 μM BMS-626529/temsavir/2% DMSO) for BMS-treated cells and 2% DMSO/PBS for PBS-treated cells. Between washes, plates were inverted and flicked to remove wash buffers and blotted briefly on paper towels. Cells were then stained with phycoerythrin-conjugated anti-human-IgG detection antibody, incubated for 30 min at room temperature, washed 3 times with wash buffer, and fixed in 0.5% formaldehyde in PBS. During all staining and washing steps, BMS-treated cells were maintained in 10 μM BMS-626529/temsavir/2% DMSO/PBS, and PBS-treated cells were maintained in 2%DMSO/PBS.

### Staining JR-FL–expressing cells enriched for smFRET State 2 with PGT151 Fab

Staining was carried out as with BMS-treated cells with the following exception. For enrichment in smFRET State 2, cells were incubated with either 50 μg/ml PGT151 Fab or with PBS for 30 min at room temperature. LIVE/DEAD Fixable Violet Dead Cell Stain staining, IgG staining, and detection antibody staining and washes were carried out as described above.

### Staining JR-FL–expressing cells enriched for smFRET State 3 with 17b Fab + sCD4

Staining was carried out as with BMS-treated cells with the following exception. For enrichment in smFRET State 3, cells were incubated with either 50 μg/ml 17b Fab +1600 nM sCD4 or with PBS for 30 min at room temperature. LIVE/DEAD Fixable Violet Dead Cell Stain staining, IgG staining, and detection antibody staining and washes were carried out as described above.

### Flow cytometry

Data were collected on a Becton Dickinson LSRFortessaTM X-50 instrument outfitted with a neutral density 1.0 filter in the PE detector. Data were analyzed using FlowJo v.9 software, and median fluorescence intensities (MFIs) were plotted against antibody concentration for calculation of AUC in Prism software.

### Calculation of ABR

All cell surface Env antibody binding experiments shown in Figure 2, Figure 3, Figure 4, Figure 5 and Figs. S2 and S4–S6 were performed in duplicate for every readout antibody concentration tested. In each plate, eight wells of cells pretreated with State-enriching ligand or with PBS received no readout antibody to determine background anti-human IgG secondary (detection) antibody binding in the absence of readout IgG (a “background binding control”). For each duplicate pair of data, the MFIs of each pair were averaged and then background-corrected by subtracting the averaged MFIs for the “background binding control” wells. MFIs were plotted against readout antibody concentration, and the AUC for each experimental group was calculated using Prism software.

Raw MFI readings varied considerably from experiment to experiment, which is attributable to differing levels of JR-FL Env expressed on the 293T cells. Therefore, we normalized the data in each experiment by calculating the ABR, defined as the ratio of the AUC in the presence of State-enriching ligand to the AUC in the absence of the ligand (PBS treatment). We found that these calculated ABRs were remarkably consistent between experiments and therefore used the ABRs to compare the binding of Abs to Env stabilized by the different State-enriching ligands.

### RMSD analysis

The RMSDs of antibody epitopes on superimposed structures were calculated by The PyMOL Molecular Graphics System, Version 2.4.0, Schrödinger, LLC (2020). Only atoms that have the same residue numbers, residue names, atom numbers, and atom names in both epitopes were included in the calculation. The epitope residues of antibodies were determined in the manner of collecting the residues with nonzero difference of solvent accessible surface area between antibody–antigen complex and antigen structures. FreeSASA was used to estimate the solvent accessible surface area (59).

## Data availability

All data described are contained within this manuscript.

## Supporting information

This article contains supporting information.

## Conflict of interest

The authors declare no competing interests with the contents of this article.
