# Supplementary material for: Antigenic analysis of the HIV-1 envelope trimer implies small differences between structural states 1 and 2
Source: J Biol Chem. 2022 Mar 10;298(4):101819. doi: 10.1016/j.jbc.2022.101819 (PMC9006658; doi:10.1016/j.jbc.2022.101819)
Supplement: Supplemental Figures S1–S9 and Table S1 [file mmc1.pdf]

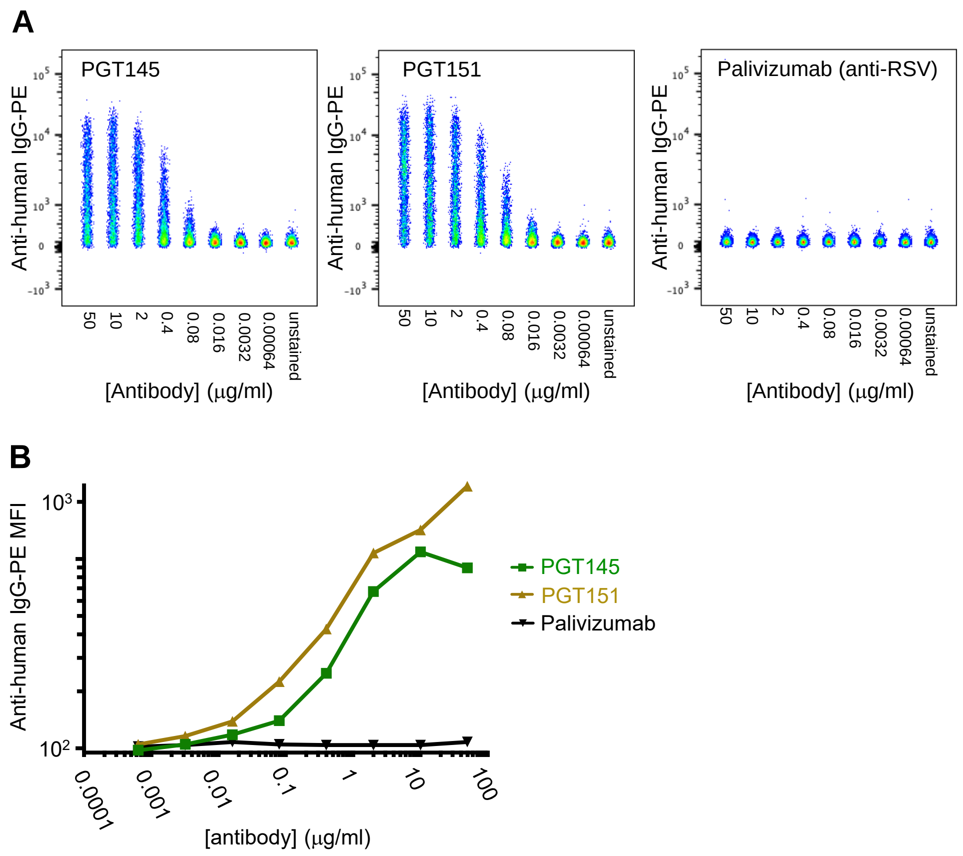

Figure S1. 293T-expressed JR-FL.JB trimers exhibit proper cleavage and native conformation based on PGT151, PGT145, and A32 binding. (A) Binding of serially diluted readout IgGs (indicated at the top of each plot) to 293T cell surface-expressed JRFL.JB Env trimers. Palivizumab, an antibody specific for respiratory syncytial virus (RSV), was used as a negative control for non-HIV-specific binding. Readout antibody binding was detected using a PE-labeled anti-human IgG secondary antibody. (B) The median fluorescence intensity (MFI) of anti-human-IgG detection antibody is plotted against each concentration of readout antibody for data displayed in (A).

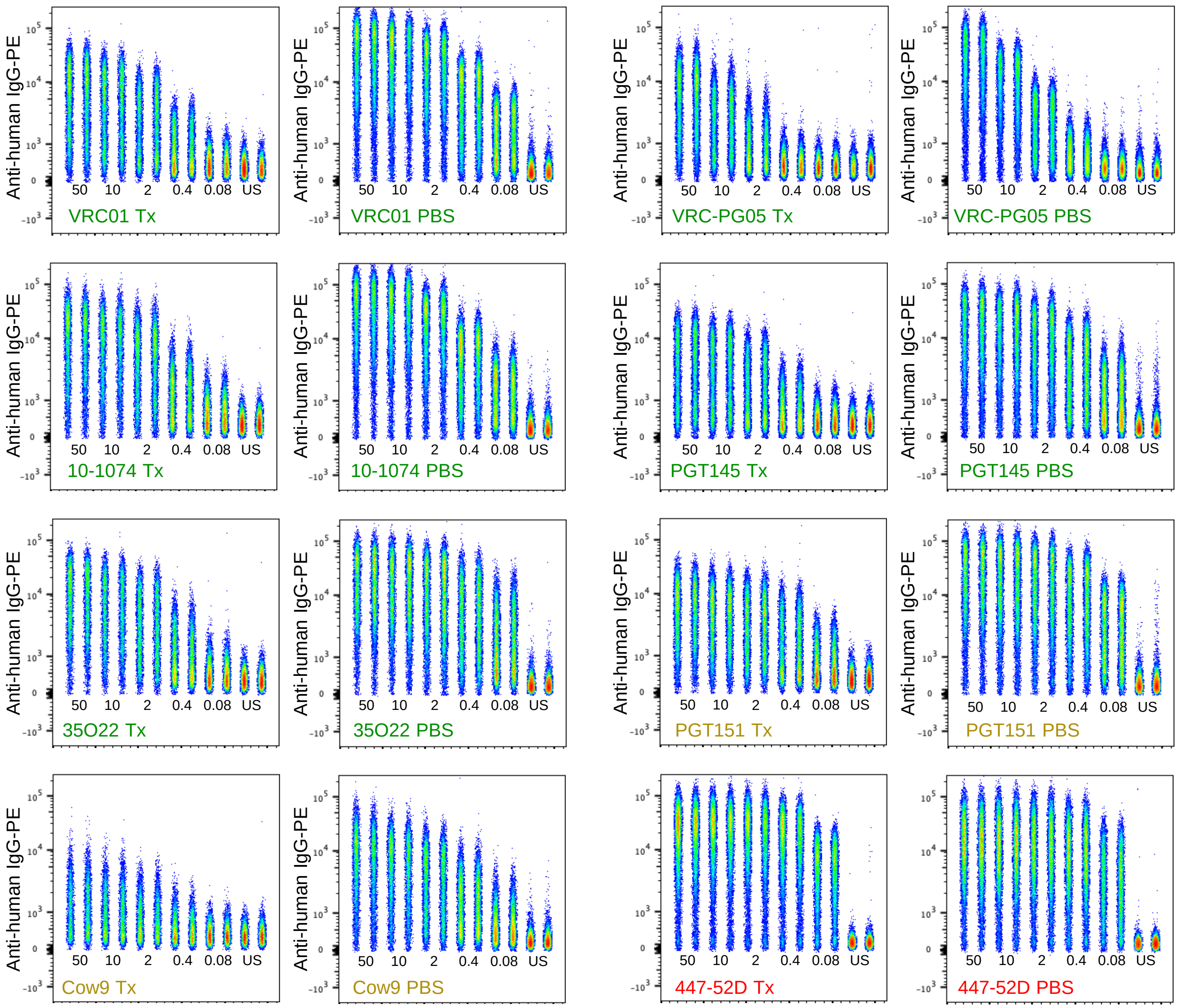

Figure S2. Enrichment of HIV-1 Env trimers in State 3 using sCD4 and 17b Fab reduced binding of State 1- and State 2-preferring antibodies. Raw flow cytometry data for Figure 2A are shown. Binding of serially diluted HIV-1-specific bnAb readout IgGs (indicated at the bottom of each plot) to 293T cell surface-expressed, fully cleaved JRFL.JB Env trimers pre-treated with soluble CD4 and 17b Fab (treated - Tx) or with PBS are shown. Readout antibody binding was detected using a PE-labeled anti-human IgG secondary antibody. In each plot, experimental duplicates are displayed, and readout antibody concentrations (μg/ml) are indicated beneath each pair of duplicates. US, unstained with readout antibody. State 1-, 2-, and 3-preferring antibodies are indicated in green, yellow, and red font, respectively. Panels showing PGT145 binding are also shown in Figure 1D as a representative example.

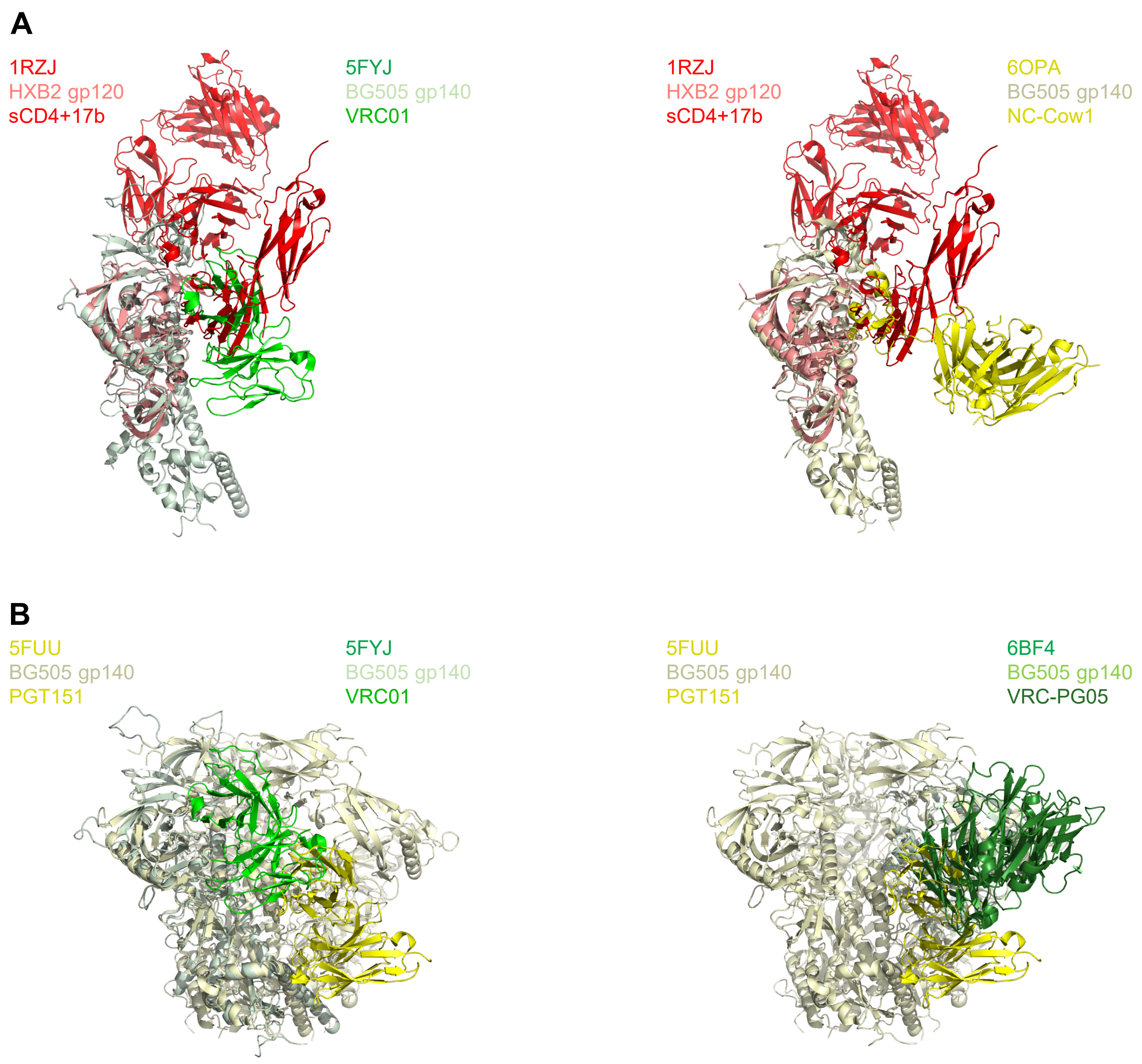

Figure S3. Structural clashes between antibodies and pre-treatment ligands. (A) PDB ID 1RZJ consisting of HIV-1 gp120 core from the HXB2 strain bound to sCD4 and 17b is colored in red in the left and right panels. (left) An overlay is shown with PDB ID 5FYJ with VRC01 shown in green with significant overlap with sCD4. (right) An overlay with PDB ID 6OPA with NC-Cow1, with a comparable binding site to Cow9, is shown in yellow with a significant overlap with sCD4. (B) PDB ID 5FUU consisting of HIV-1 Env trimer from the JR-FL strain bound to PGT151 is colored in yellow in the left and right panels. (left) An overlay is shown with PDB ID 5FYJ with VRC01 shown in green with significant overlap with PGT151. (right) An overlay is shown with PDB ID 6BF4 with VRC-PG05 is shown in dark-green with significant overlap with PGT151.

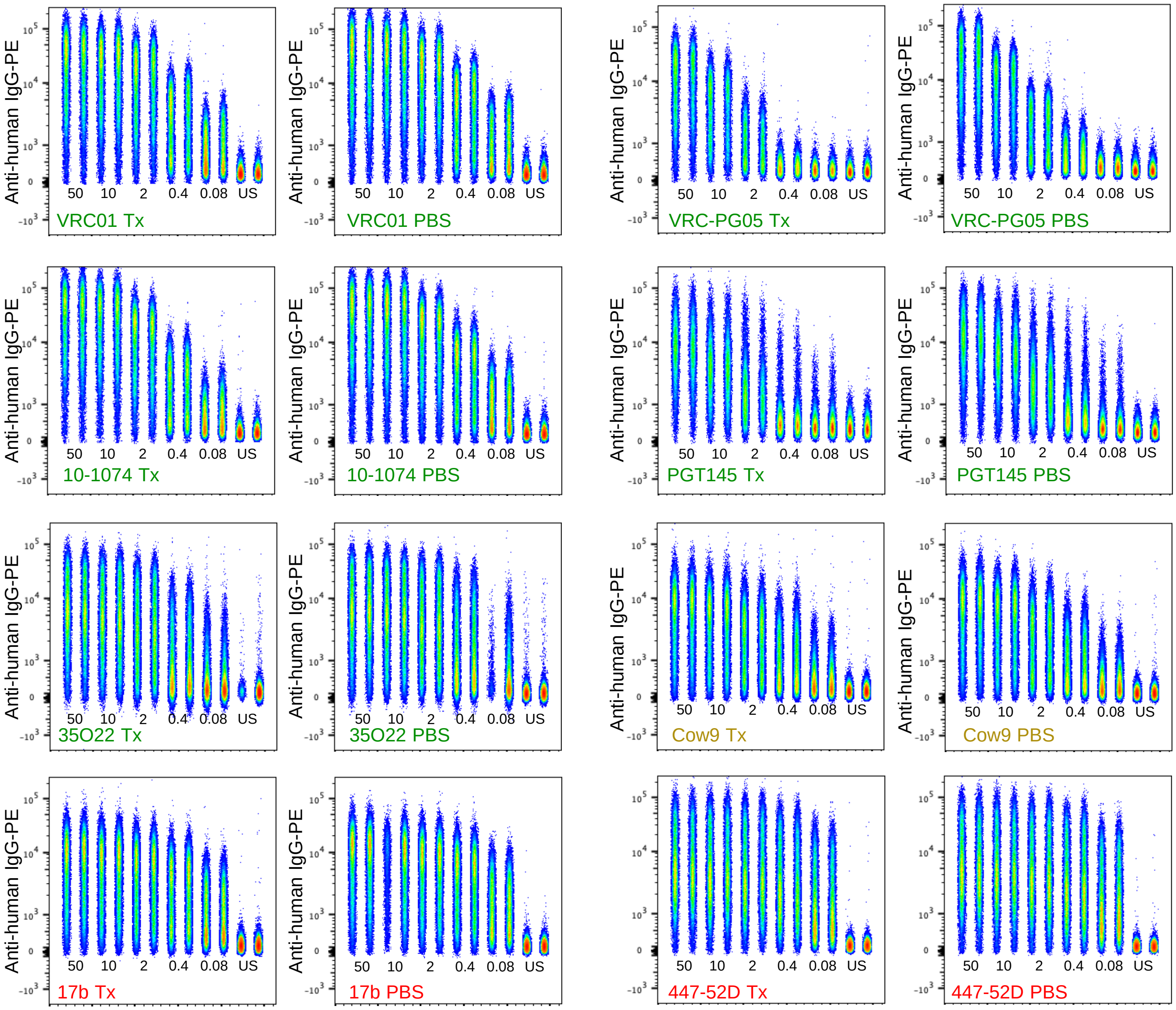

Figure S4. Enrichment of State 2 using PGT151 Fab causes a minor to moderate reduction in binding of State 1- and State 3-preferring antibodies. Raw flow cytometry data for Figure 3A are shown. Binding of serially diluted HIV-1-specific bnAb readout IgGs (indicated at the bottom of each plot) to 293T cell surface-expressed, fully cleaved JRFL.JB Env trimers pre-treated with PGT151 Fab (treated - Tx) or with PBS are shown. Readout antibody binding was detected using a PE-labeled anti-human IgG secondary antibody. In each plot, experimental duplicates are displayed, and readout antibody concentrations (μg/ml) are indicated beneath each pair of duplicates. US, unstained with readout antibody. State 1-, 2-, and 3-preferring antibodies are indicated in green, yellow, and red font, respectively.

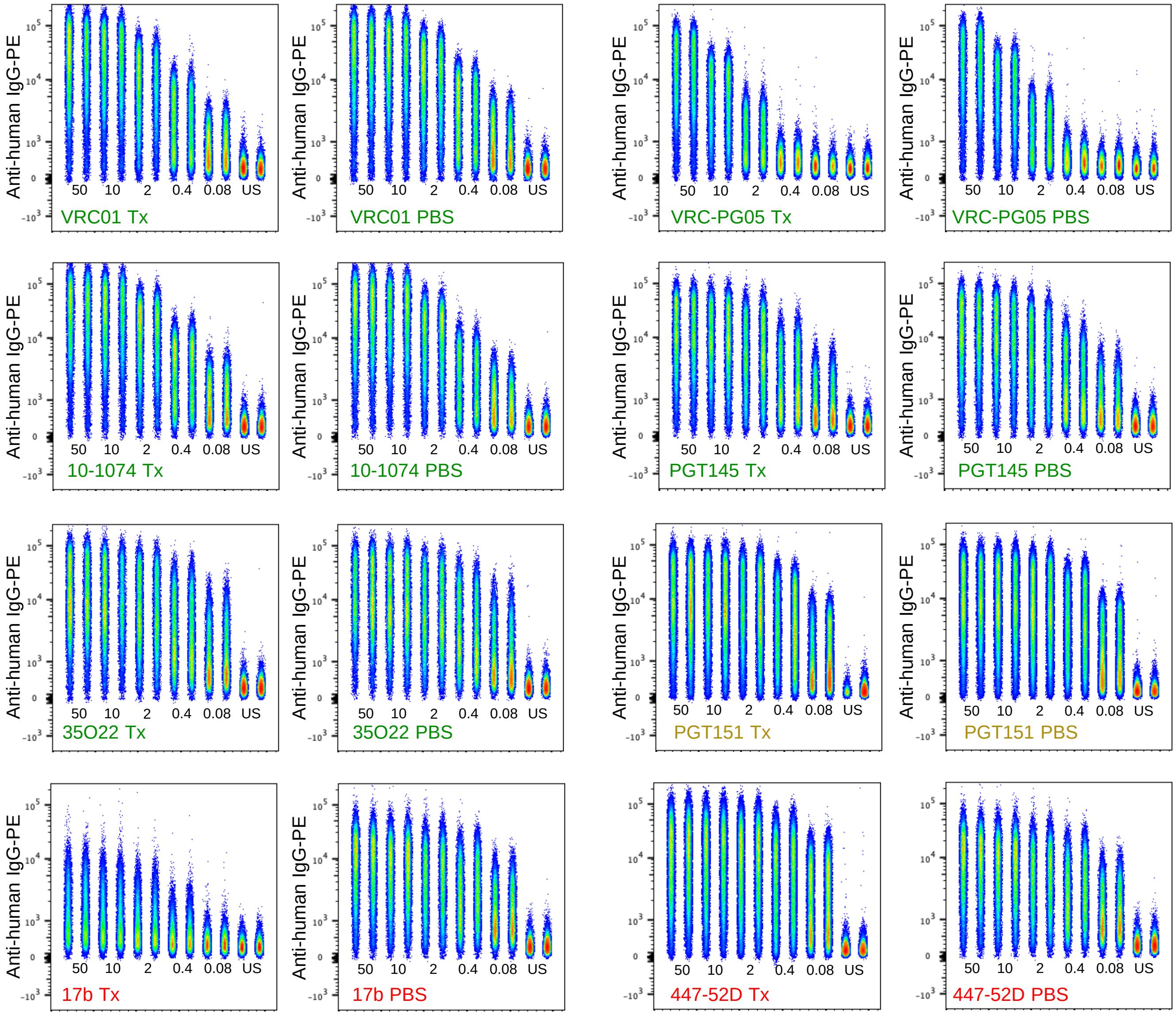

Figure S5. Enrichment of State 1 using BMS-626529/temsavir has minimal effect on binding of State 2-preferring antibodies but reduces binding of State 3-preferring antibodies. Raw flow cytometry data for Figure 4A are shown. Binding of serially diluted HIV-1-specific bnAb readout IgGs (indicated at the bottom of each plot) to 293T cell surface-expressed, fully cleaved JRFL.JB Env trimers pre-treated with BMS-626529/temsavir (treated - Tx) or with PBS are shown. Readout antibody binding was detected using a PE-labeled anti-human IgG secondary antibody. In each plot, experimental duplicates are displayed, and readout antibody concentrations (μg/ml) are indicated beneath each pair of duplicates. US, unstained with readout antibody. State 1-, 2-, and 3-preferring antibodies are indicated in green, yellow, and red font, respectively.

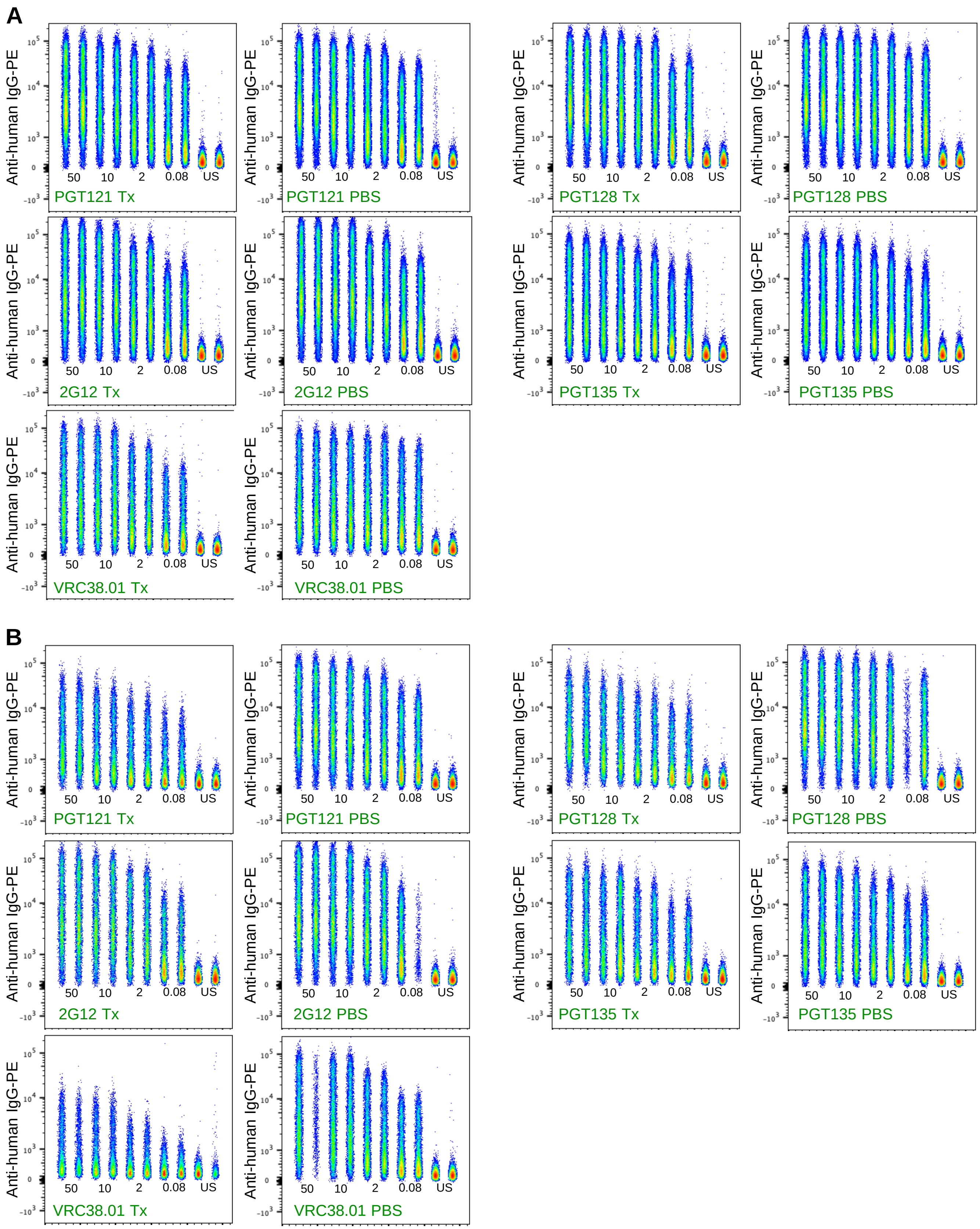

Figure S6. Enrichment of State 2 using PGT151 Fab has minimal effect on binding of State 1-preferring antibodies, while enrichment of State 3 using soluble CD4 and 17b Fab reduces binding of State 1-preferring antibodies. Shown are raw flow cytometry data used for determining ABRs in Figures 5C and 6A. (A) Binding of serially diluted HIV-1-specific bnAb readout IgGs (indicated at the bottom of each plot) to 293T cell surface-expressed, fully cleaved JRFL.JB Env trimers pre-treated with PGT151 Fab (treated - Tx) or with PBS are shown. (B) Antibody binding of readout IgGs to trimers pre-treated with soluble CD4 and 17b Fab are shown. Readout antibody binding was detected using a PE-labeled anti-human IgG secondary antibody. In each plot, experimental duplicates are displayed, and readout antibody concentrations (μg/ml) are indicated beneath each pair of duplicates. US, unstained with readout antibody. Raw data for generating State 2 and State 3 ABRs for 10-1074, PGT145, and 35O22 are shown in Figures S2 and S4.

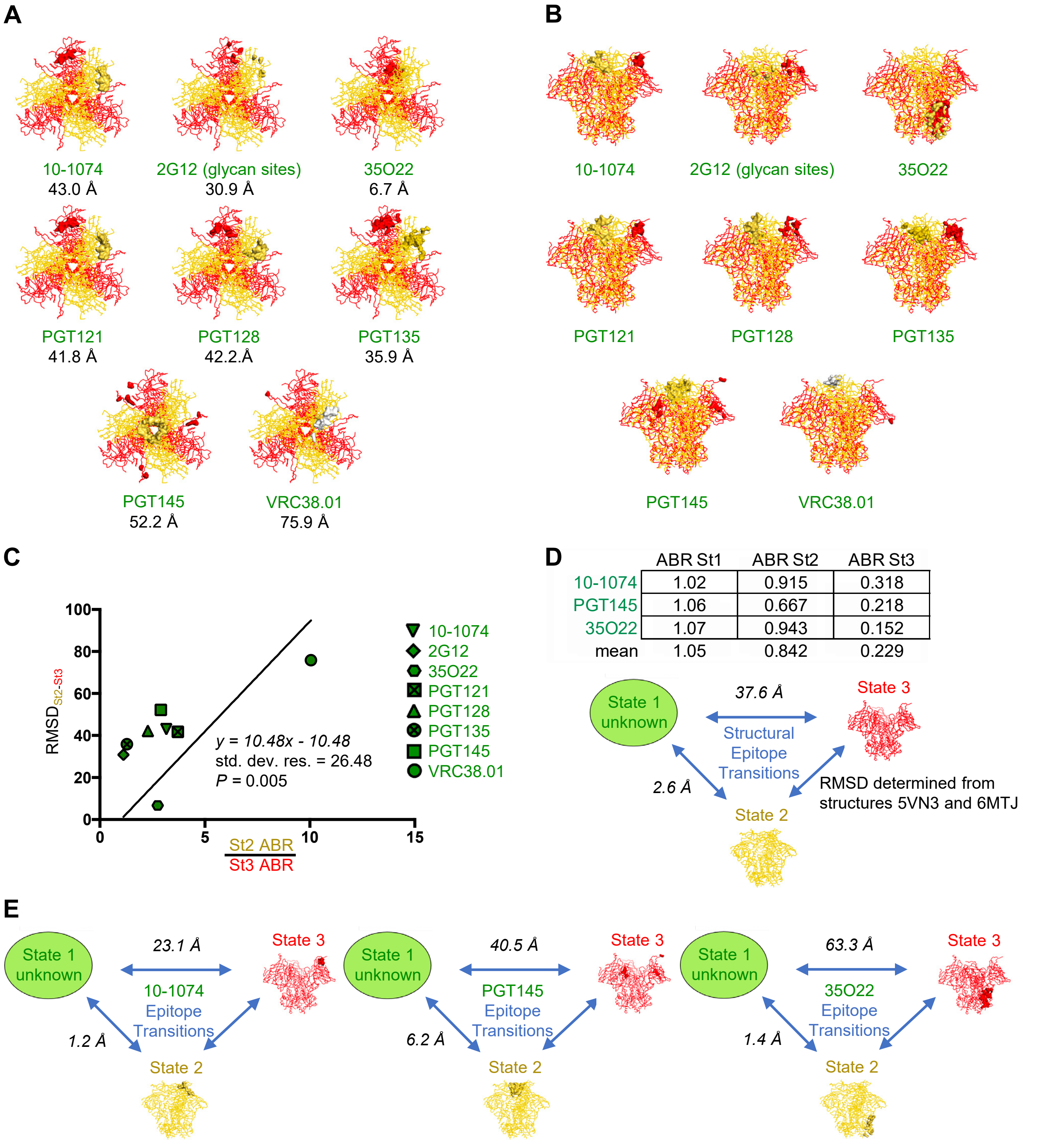

	ABR St1	ABR St2	ABR St3
10-1074	1.02	0.915	0.318
PGT145	1.06	0.667	0.218
35O22	1.07	0.943	0.152
mean	1.05	0.842	0.229

Figure S7. When available State 1 and State 2 structures are aligned by gp41, we predict State 1-preferring antibody epitope RMSDs between States 1 and 3 to be about 20-65 angstroms and RMSDs between States 1 and 2 to be about 1-6 angstroms. (A) Ribbon diagrams of the HIV-1 Env structure enriched in State 2 (yellow) and State 3 (red) from structures 6MTJ and 5VN3, respectively, are shown as aligned by gp41 as viewed from the top and (B) as viewed from the side. The epitope sites of different state-preferring antibodies are indicated by the color-coded space-filling models. While only one residue for the VRC38.01 epitope was indicated in the 6MTJ structure, the entire epitope is indicated in the white space-filling model. RMSDs between epitopes in State 2 and State 3 are indicated below each antibody name in panel A. (C) The RMSD of different nAb-bound epitope sites (indicated in different symbols) in State 2 and State 3, as determined from solved structures 5VN3 and 6MTJ aligned by gp41, is plotted against the ratio of State 2 ABRs and State 3 ABRs measured for different nAbs in the binding assay, and a Pearson correlation was calculated when forcing the *x* and *y* intercepts through (1,0). (D) The mean ABRs of States 1, 2, and 3 are shown for State 1-preferring antibodies 10-1074, PGT145, and 35O22. The mean RMSDs (italicized font) of nAb epitope sites between States 1 and 2, and between States 1 and 3, were calculated from the ratios of the mean ABRs (as calculated in Figure 6A) and the Pearson correlation shown in Panel C, where *y* is the RMSD and *x* is the ratio of ABRs. (E) The same analysis shown in Panel D was performed for the individual antibodies 10-1074, PGT145, and 35O22.

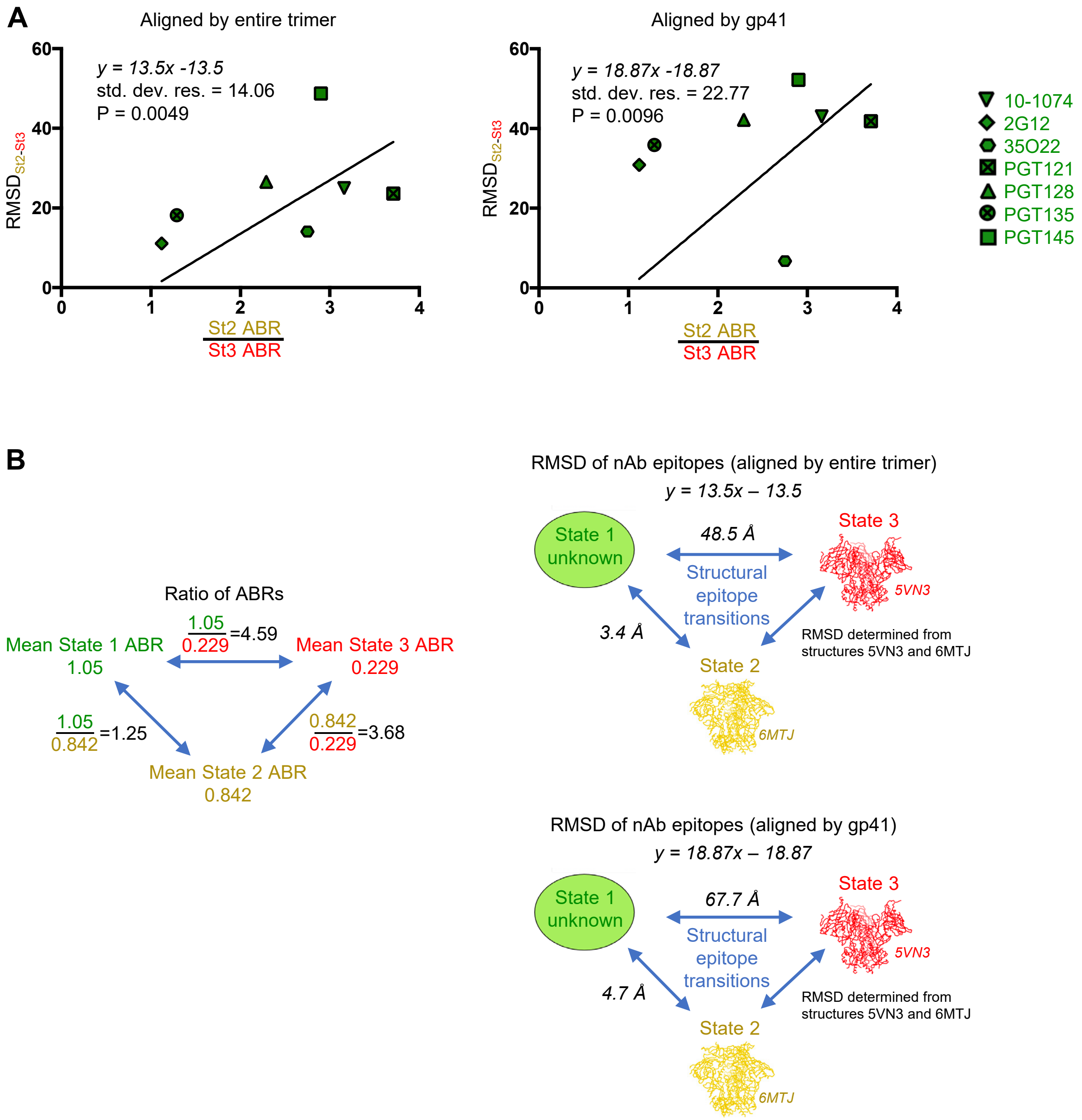

Figure S8. Based on ratios of ABRs and available structural data (removing the outlier VRC38.01), we predict State 1-preferring antibody epitope RMSDs between States 1 and 3 to be about 50-70 angstroms and RMSDs between States 1 and 2 to be about 3-5 angstroms. (A) The RMSD of different nAb-bound epitope sites (indicated in different symbols) in State 2 and State 3, as determined from solved structures 5VN3 and 6MTJ aligned by the entire Env trimer and by gp41 alone, is plotted against the ratio of State 2 ABRs and State 3 ABRs measured for different nAbs in the binding assay, and a Pearson correlation was calculated when forcing the *x* and *y* intercepts through (1,0). The outlier VRC38.01 was removed for this analysis. (B) The ratios of ABRs between State 1 and State 2, between State 1 and State 3, and between State 2 and State 3 were calculated for multiple antibodies using the HIV-1 Env cell surface staining assay. Shown in black font are the ratios of the mean of ABRs for 10-1074, 35O22, and PGT145, the three bnAbs from which we could calculate ABRs to trimers stabilized by each of the three states. On the right, the mean RMSDs (italicized font) of nAb epitope sites between States 1 and 2 and between States 1 and 3 were calculated from the ratios *x* of the measured ABRs shown on the left and the Pearson correlation equations (shown) derived in panel A, where *y* is the RMSD and *x* is the ratio of ABRs.

A

	ABR St1	ABR St2	ABR St3
10-1074	1.02	0.915	0.318
PGT145	1.06	0.667	0.218
35O22	1.07	0.943	0.152

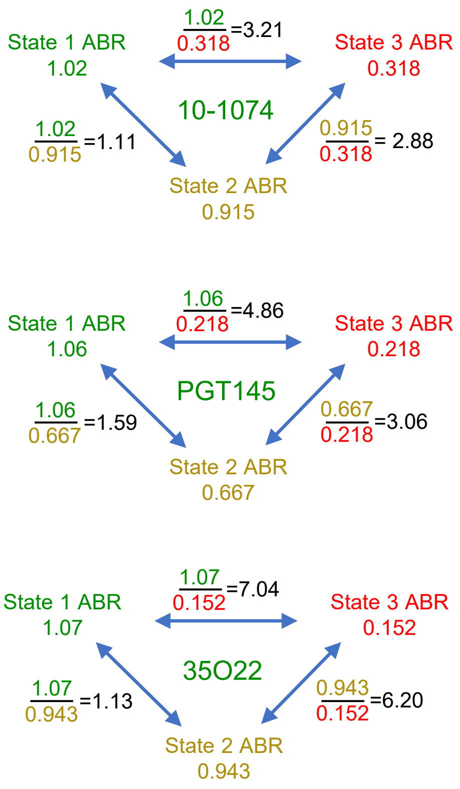

B

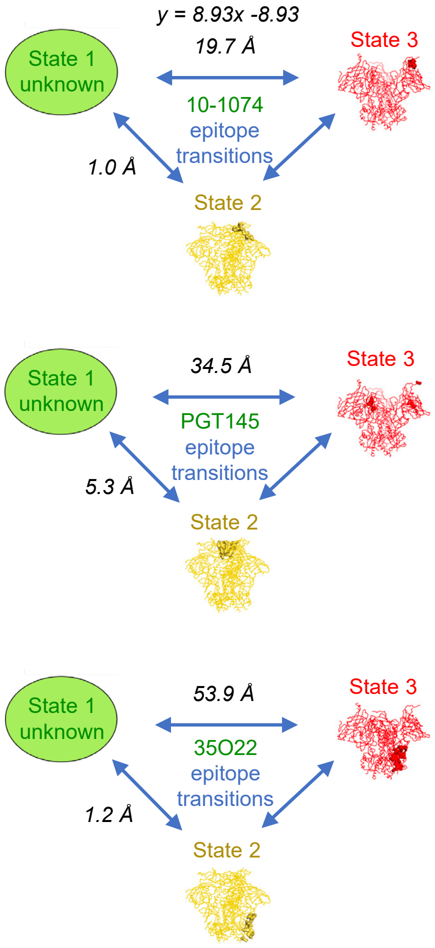

Figure S9. Based on ratios of ABRs and available structural data, we predict State 1-preferring antibody epitope RMSDs between States 1 and 3 to be about 20-55 Å and RMSDs between States 1 and 2 to be about 1-5 Å. (A) The State 1, State 2, and State 3 ABRs for State 1-preferring antibodies 10-1074, PGT145, and 35O22 are shown, and the ratios of those ABRs are calculated below. (B) The RMSDs (italicized font) of nAb epitope sites between States 1 and 2 and between States 1 and 3 were calculated from the ratios *x* of the measured ABRs shown in panel A and the Pearson correlation equation (shown) derived in Figure 5B, where *y* is the RMSD and *x* is the ratio of ABRs.

Table S1. Epitope targets and state preferences of antibodies used in the study.

Epitope	Antibody	State preference
CD4bs	VRC01	1
	Cow9	2
V2 apex	PGT145	1
	VRC38.01	1
Outer domain glycan	2G12	1
glycan-V3	10-1074	1
	PGT121	1
	PGT128	1
	PGT135	1
silent face center	VRC-PG05	1
gp120:gp41 interface	35O22	1
	PGT151	2
CD4-induced epitope	17b	3
V3 loop	447-52D	3
